# Supplementary material for: Time-Dependent Changes in Hepatic Sphingolipid Accumulation and PI3K/Akt/mTOR Signaling Pathway in a Rat Model of NAFLD
Source: Int J Mol Sci. 2021 Nov 19;22(22):12478. doi: 10.3390/ijms222212478 (PMC8618899; doi:10.3390/ijms222212478)

Figure S1. The total expression of (A) glucose transporter 2 (GLUT2) in the liver homogenates. The protein expression was measured using the Western blot technique and presented as a percentage difference compared to the control group, which was set as 100%. The data are presented as mean ± SD and based on n = 6 rats per group in each week of the high-fat diet (HFD). * p < 0.05 significant difference vs. control group (0 week).

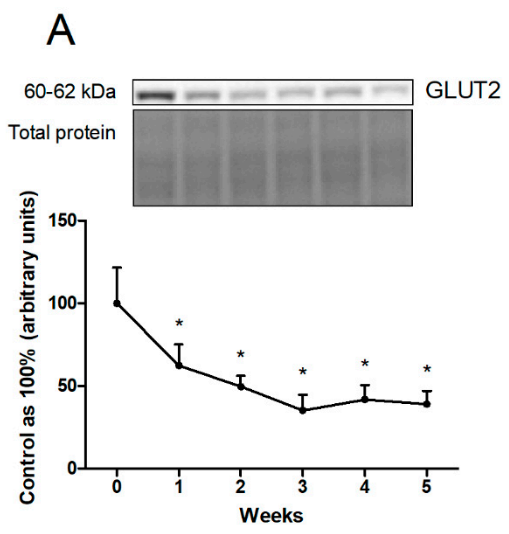

Supplement: Supplementary file 1 [file ijms-22-12478-s001.zip › ijms-1453102-supplementary.pdf]
